# Supplementary material for: Self-Assembled Permanent Micro-Magnets in a Polymer-Based Microfluidic Device for Magnetic Cell Sorting
Source: Cells. 2021 Jul 9;10(7):1734. doi: 10.3390/cells10071734 (PMC8307954; doi:10.3390/cells10071734)
Supplement: Supplementary file 1 [file cells-10-01734-s001.zip › cells-1255033 supp.pdf]

Supplementary Materials

Self-assembled permanent micro-magnets in a polymer-based microfluidic device for magnetic cell sorting

Lucie Descamps [1], Marie-Charlotte Audry [1], Jordyn Howard [1], † Samir Mekkaoui [1], Clément Albin [2], David Barthelemy [3], Léa Payen [3], Jessica Garcia [3], Emmanuelle Laurenceau [4], Damien Le Roy [2] and Anne-Laure Deman[2,*]

1 Univ Lyon, Université Claude Bernard Lyon 1, CNRS, INSA Lyon, Ecole Centrale de Lyon, CPE Lyon, INL, UMR5270, 69622 Villeurbanne, France
2 Institut Lumière Matière ILM-UMR 5306, CNRS, Université Lyon 1, Villeurbanne F-69622, France
3 Hospices Civils de Lyon, Centre Hospitalier Lyon–Sud, Biochemistry, Pharmacotoxicology, and Molecular Biology Department, Université Lyon 1, Pierre Bénite, France
4 Univ Lyon, Ecole Centrale de Lyon, CNRS, INSA Lyon, Université Claude Bernard Lyon 1, CPE Lyon, CNRS, INL, UMR5270, 69130 Ecully, France
* Correspondence: anne-laure.deman-him@univ-lyon1.fr;

This PDF file includes:

Figure S1. SEM image of NdFeB particles Page 2

Figure S2. Flowchart of the micro-magnet fabrication Page 3

Figure S3. Approach/retract curve from colloidal probe AFM measurement Page 4

Figure S4. Picture of the experimental set-up Page 5

Figure S5. Magnetization curve of the superparamagnetic beads Page 6

Figure S6. Magnetization curve of the NdFeB particles Page 7

Equation and numerical simulation of the magnetic field gradient Page 8

Figure S7. Numerical simulations of a network of increasing chain number Page 9

Figure S8. SEM image of superparamagnetic beads Page 10

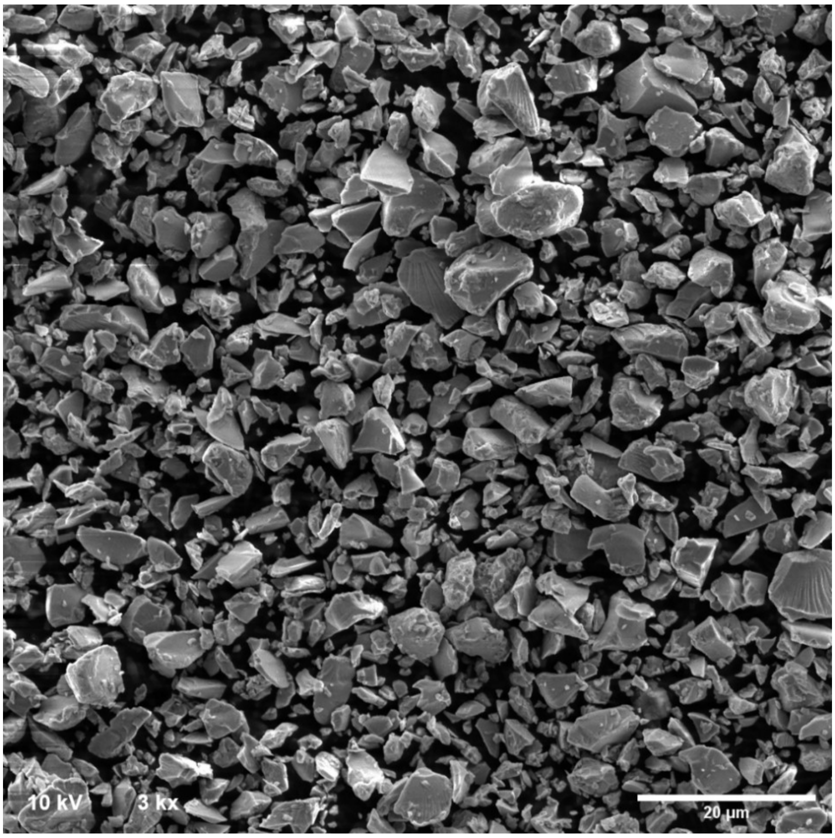

Figure S1. SEM image of irregularly shaped crushed melt-spun ribbon NdFeB micro-particles (0.5-7 μm size) that were supplied by Magnequench International, Inc.

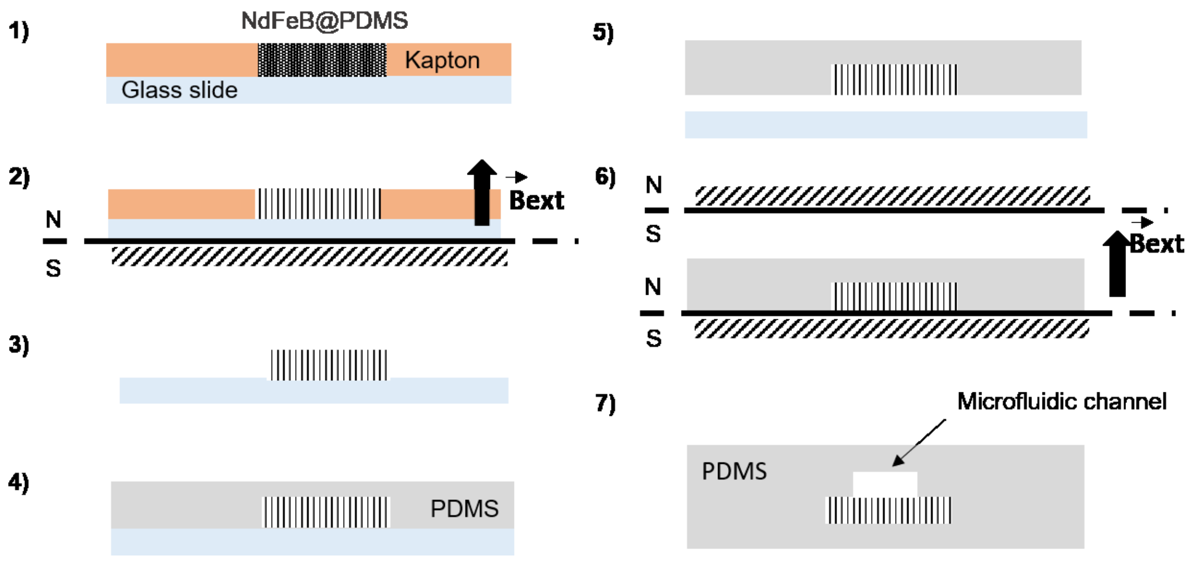

Figure S2. Fabrication steps of the microdevice: 1) the composite is molded in a Kapton film bonded to a silanized glass slide substrate. 2) The composite is then placed in a 300 mT magnetic field for NdFeB particles self-organization in chains at 60°C for 2h. 3-4) The Kapton mold is then removed and pure PDMS is poured. 5) After curing at 70°C for 2h, the composite membrane is peeled off and 6) magnetized under a magnetic field of 1 T. Finally, 7) the composite membrane is bonded to a channel molded in PDMS by O_2 surface plasma activation.

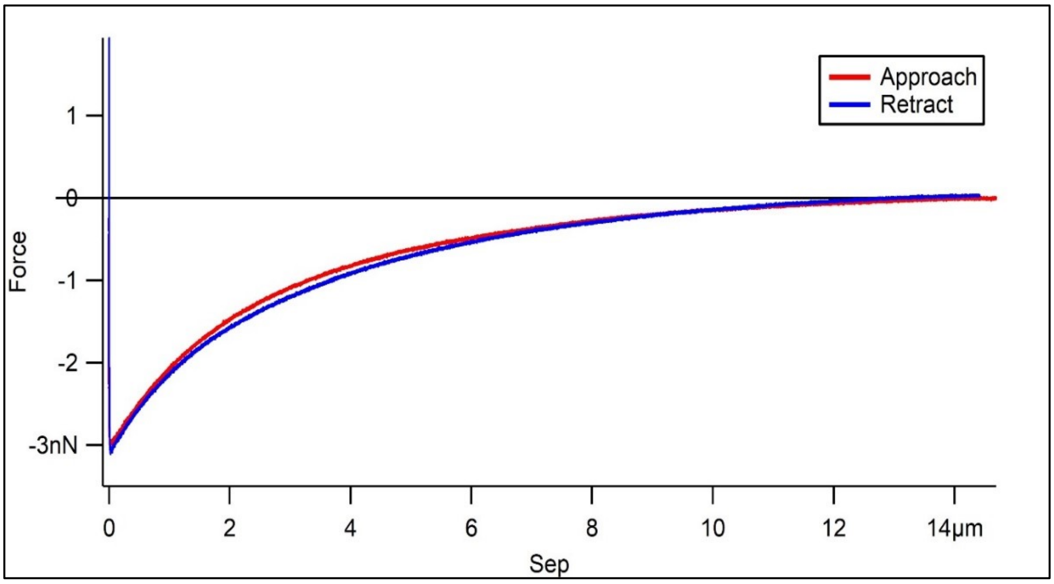

Figure S3. Approach/retract curve obtained by colloidal probe AFM measurements with a constant velocity of 1 µm/s, at the exact micro-magnet position, in presence of the external millimeter-sized magnet.

The sample surface is scanned in two-pass mode. The first scan, at contact, is performed to localize the micro-magnets; the second scan, at 500 nm above the sample surface, records the cantilever's deflection and measures the maximum force generated by a localized micro-magnet, thanks to the recorded approach/retract curves.

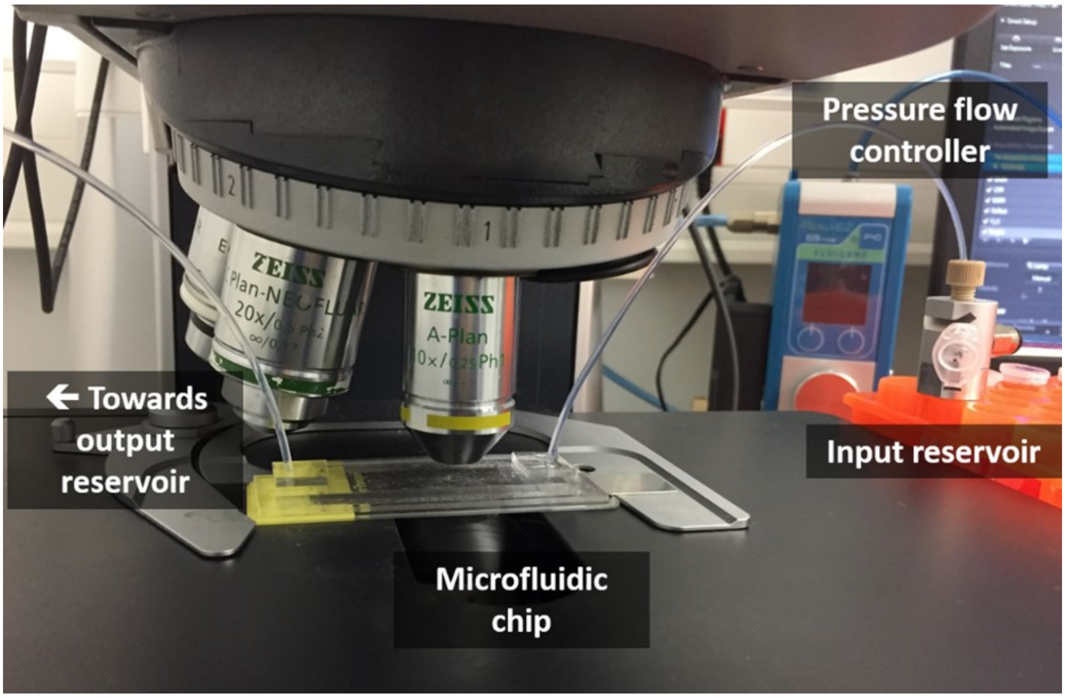

Figure S4. Picture of the experimental set up: the microfluidic system is placed under the microscope. Inlet tank and pressure driven injection system Flow EZ™ are from Fluigent®.

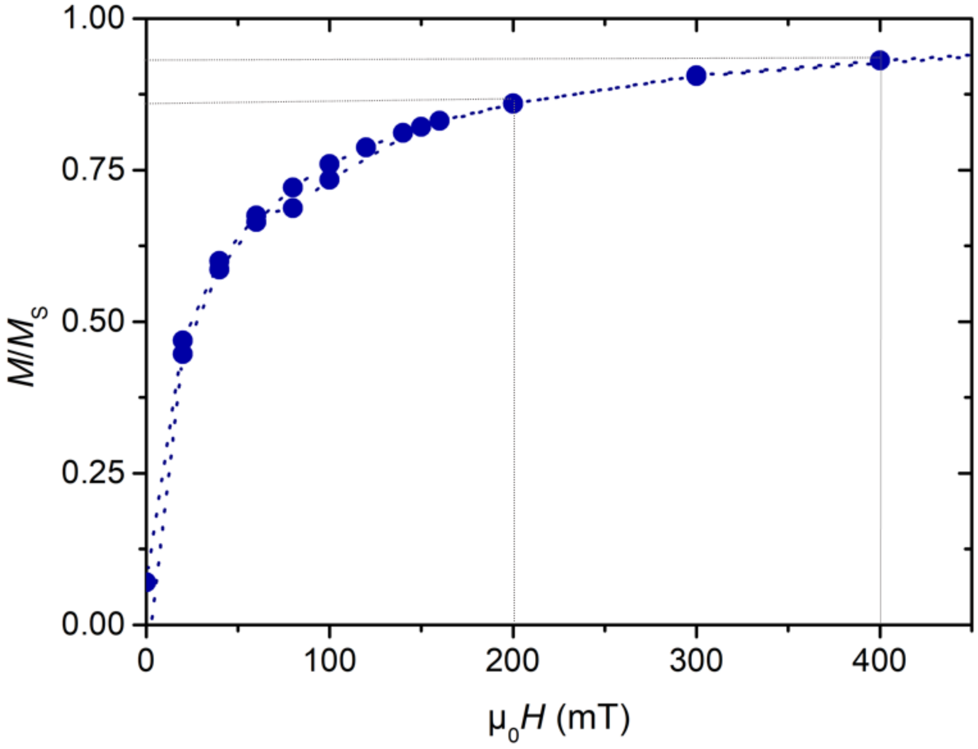

Figure S5. Normalized magnetization curve at 300K of superparamagnetic beads, obtained by SQUID magnetometer. M_S = 550 kA/m. In the absence of an external magnet, the micro-magnets are submitted to a magnetic field of 200 mT. In the presence of the external magnet, the applied magnetic field is doubled, and reaches 400 mT.

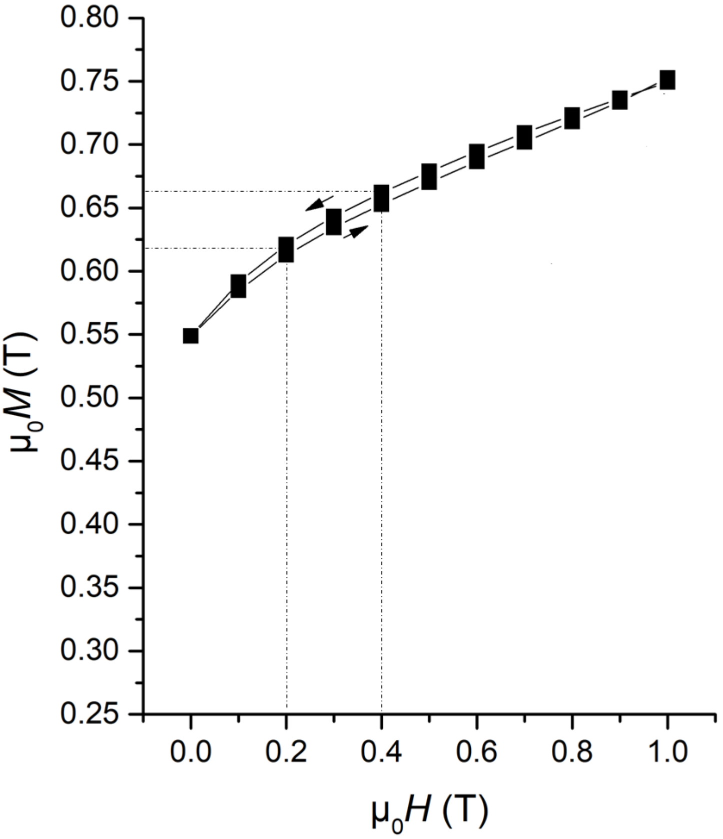

Figure S6. Magnetization curve of NdFeB particles, obtained by SQUID magnetometer. In the absence of an external magnet (μ_0H=200 mT), the particles generate a field of 0.62 T, corresponding to a magnetization of 490 kA/m. In the presence of the external magnet (μ_0H=400 mT), they produce a field of 0.67 T, corresponding to a magnetization of 515 kA/m.

Supplementary: Equation and numerical simulation of the magnetic field gradient

For a particle of volume V_p, with a saturation magnetization M_{sp}, exposed to a magnetic field B, the magnetic force applied on the particle can be expressed as follows:

$$\overrightarrow{F_{mag}} = V_p(\overrightarrow{M_{sp}} \cdot \vec{\nabla})\vec{B}$$

The expression of the magnetic force was developed so as to identify a magnetic coefficient, C_m, which pictures the magnetic field gradient.

$$\overrightarrow{F_{mag}} = V_p M_{sp} \left(\frac{\vec{B}}{\|\vec{B}\|} \cdot \vec{\nabla} \right) \vec{B}$$

$$\overrightarrow{F_{mag}} = \frac{V_p M_{sp}}{\|\vec{B}\|} \begin{pmatrix} B_r \\ B_z \end{pmatrix} \cdot \begin{pmatrix} \frac{\partial}{\partial r} \\ \frac{\partial}{\partial z} \end{pmatrix} \begin{pmatrix} B_r \\ B_z \end{pmatrix}$$

$$\overrightarrow{F_{mag}} = \frac{V_p M_{sp}}{\|\vec{B}\|} \left(B_r \frac{\partial}{\partial r} + B_z \frac{\partial}{\partial z} \right) \begin{pmatrix} B_r \\ B_z \end{pmatrix}$$

$$\overrightarrow{F_{mag}} = \frac{V_p M_{sp}}{\sqrt{{B_r}^2 + {B_z}^2}} \begin{pmatrix} B_r \frac{\partial B_r}{\partial r} + B_z \frac{\partial B_r}{\partial z} \\ B_r \frac{\partial B_z}{\partial r} + B_z \frac{\partial B_z}{\partial z} \end{pmatrix} \begin{matrix} \overrightarrow{e_r} \\ \overrightarrow{e_z} \end{matrix}$$

We considered the vertical component of the magnetic field gradient, and the vertical magnetic coefficient $C_{m,z}$ was calculated as follows:

$$Cm, z = \frac{1}{\sqrt{{B_r}^2 + {B_z}^2}} \left(B_r \frac{\partial B_z}{\partial r} + B_z \frac{\partial B_z}{\partial z} \right)$$

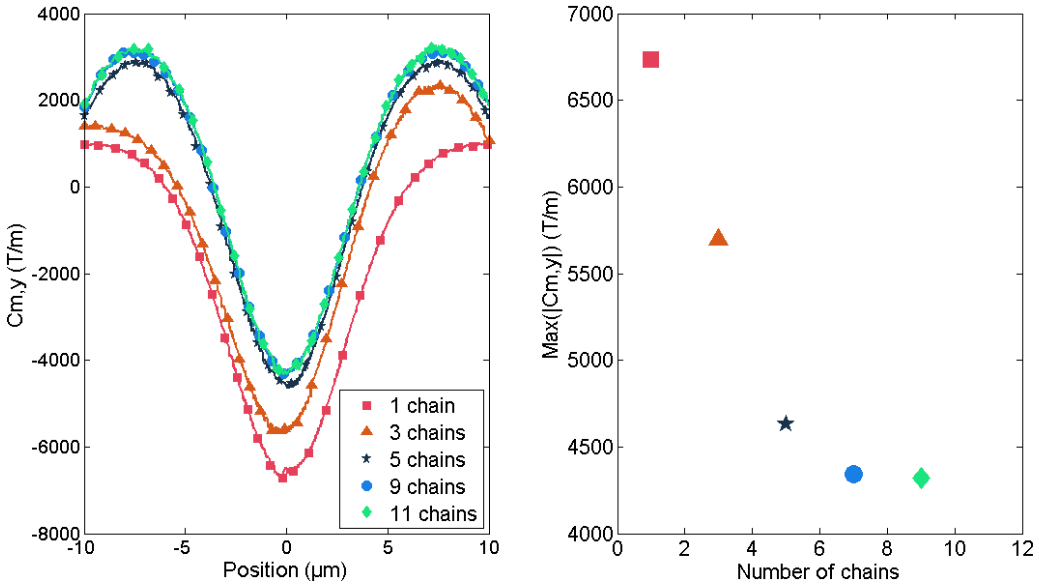

Figure S7. Numerical simulations of the magnetic coefficient Cm,y for an increasing number of chains in the array. Cm,y was calculated above the central micro-magnet of the network.

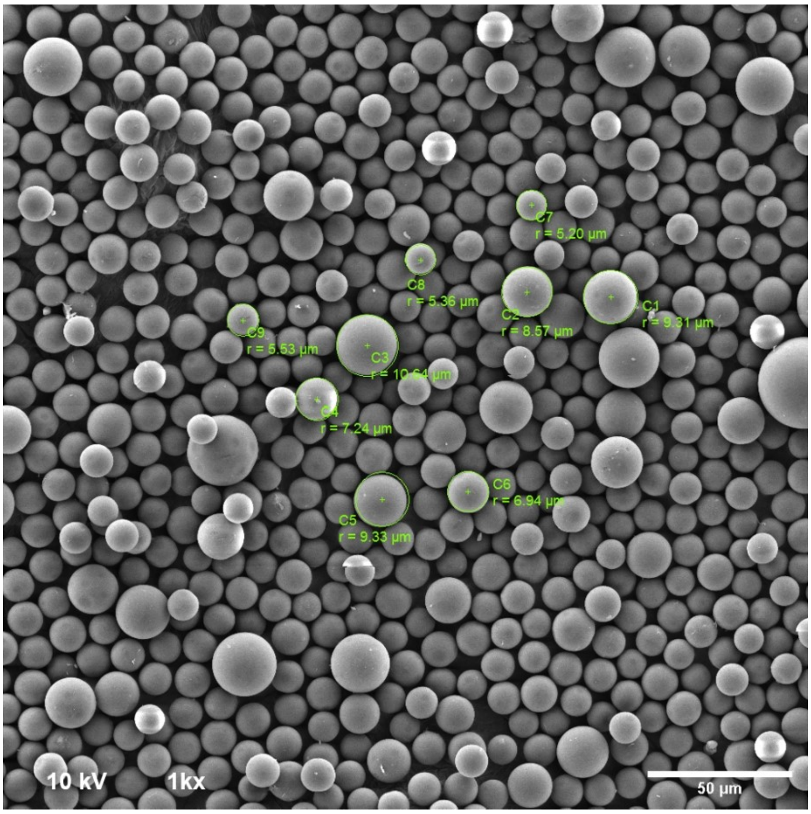

Figure S8. SEM image of superparamagnetic beads (average diameter: 12 µm, density: 1.1 g/cm3, magnetization: 0.55 kA/m, material: magnetite nano-inclusions in a polystyrene matrix, 1 vol %Fe3O4), purchased from Kisker. The bead radius varies from 4 to 10 µm.
